# Five-year illness trajectories across racial groups in the UK following a first episode psychosis

**DOI:** 10.1007/s00127-023-02428-w

**Published:** 2023-01-30

**Authors:** Siân Lowri Griffiths, Tumelo Bogatsu, Mia Longhi, Emily Butler, Beel Alexander, Mrunal Bandawar, Linda Everard, Peter B. Jones, David Fowler, Joanne Hodgekins, Tim Amos, Nick Freemantle, Paul McCrone, Swaran P. Singh, Max Birchwood, Rachel Upthegrove

**Affiliations:** 1 Institute for Mental Health, University of Birmingham, Edgbaston, Birmingham, B15 2TT UK; 2 Birmingham and Solihull Mental Health Foundation Trust, Birmingham, UK; 3 Department of Psychiatry, University of Cambridge and CAMEO, Cambridge and Peterborough NHS Foundation Trust, Fulbourn, UK; 4 Department of Psychology, University of Sussex, Brighton, UK; 5 Norwich Medical School, University of East Anglia, Norwich, UK; 6 Academic Unit of Psychiatry, University of Bristol, Bristol, UK; 7 Institute of Clinical Trials and Methodology, University College London, London, UK; 8 Institute for Life Course Development, University of Greenwich, London, UK; 9 Mental Health and Wellbeing Warwick Medical School, University of Warwick, Coventry, UK

**Keywords:** Outcomes, Early psychosis, Ethnicity, Deprivation, Inequities

## Abstract

**Purpose:**

Psychosis disproportionally affects ethnic minority groups in high-income countries, yet evidence of disparities in outcomes following intensive early intervention service (EIS) for First Episode Psychosis (FEP) is less conclusive. We investigated 5-year clinical and social outcomes of young people with FEP from different racial groups following EIS care.

**Method:**

Data were analysed from the UK-wide NIHR SUPEREDEN study. The sample at baseline (*n* = 978) included White (*n* = 750), Black (*n* = 71), and Asian (*n* = 157) individuals, assessed during the 3 years of EIS, and up to 2 years post-discharge (*n* = 296; Black [*n* = 23]; Asian [*n* = 52] and White [*n* = 221]). Outcome trajectories were modelled for psychosis symptoms (positive, negative, and general), functioning, and depression, using linear mixed effect models (with random intercept and slopes), whilst controlling for social deprivation. Discharge service was also explored across racial groups, 2 years following EIS.

**Results:**

Variation in linear growth over time was accounted for by racial group status for psychosis symptoms—positive (95% CI [0.679, 1.235]), negative (95% CI [0.315, 0.783]), and general (95% CI [1.961, 3.428])—as well as for functioning (95% CI [11.212, 17.677]) and depressive symptoms (95% CI [0.261, 0.648]). Social deprivation contributed to this variance. Black individuals experienced greater levels of deprivation (*p* < 0.001, 95% CI [0.187, 0.624]). Finally, there was a greater likelihood for Asian (OR = 3.04; 95% CI [2.050, 4.498]) and Black individuals (OR = 2.47; 95% CI [1.354, 4.520]) to remain in secondary care by follow-up.

**Conclusion:**

Findings suggest variations in long-term clinical and social outcomes following EIS across racial groups; social deprivation contributed to this variance. Black and Asian individuals appear to make less improvement in long-term recovery and are less likely to be discharged from mental health services. Replication is needed in large, complete data, to fully understand disparities and blind spots to care.

**Supplementary Information:**

The online version contains supplementary material available at 10.1007/s00127-023-02428-w.

## Introduction

The incidence of psychosis disproportionally affects ethnic minority groups in high-income countries [1]. Black Caribbean individuals are five times more likely to develop psychosis in the UK, compared to the White British population, but such incidence rates are not mirrored in Caribbean countries [2]. Further, for individuals of Pakistani, Bangladeshi, or of Mixed ethnic backgrounds in England, the incidence rates are twice as high compared to the White population [1].

It is well documented that inequalities exist in access to mental health care, for example, Black individuals are more likely to experience adverse pathways to care [3]. Differences may also exist in the type of care offered and received by ethnic minorities within mental health services. Black Caribbean and Black African individuals with psychosis are 15–30% less likely to receive Cognitive–Behavioural Therapy (CBT) compared to White individuals with psychosis in the UK [4]. National clinical audit data has also highlighted inequalities in the offer of clozapine; Black individuals are up to 44% less likely to be offered this evidenced-based medication for treatment-resistant psychosis [5].

Despite this, less robust and consistent research has been carried out on the impact of this potential disparity on course and outcome of psychosis [6, 7]. A systematic review has provided evidence that migrant groups are more likely to achieve remission but have higher rates of involuntary admission and disengagement compared to host populations [8, 9]. In studies comparing outcomes across ethnic groups, poorer social and clinical outcomes for Black individuals are reported compared to White individuals [6, 7, 10–15]. For other racial groups, outcomes have been reported to be more benign. For example, in an exploratory study by Birchwood et al., relapse and readmission rates were the highest for Black Caribbean individuals, and lowest in those of Asian heritage, when compared to White British individuals [10]. Family structure, quicker access to care, and employment status have been proposed to mitigate these effects [10]. However, inconsistencies and methodological constraints, such as small sample size, high attrition, short follow-up, and retrospective designs, make it difficult to draw on conclusions regarding any differences in clinical and social outcomes for ethnic minority individuals with psychosis, and questions remain over why such differences exist [7, 12].

In a more recent longitudinal study, the AESOP-10 cohort study investigated ethnic disparities in illness outcomes between Black minority ethnic and White British individuals 10 years following a first episode psychosis (FEP) [6]. Compared to the White British group, the Black Caribbean group had poorer clinical, social, and service use outcomes. There was also some initial evidence suggesting social disadvantage and isolation contributed to the differences in symptoms and social outcomes [6].

It is important to understand malleable factors and social inequalities related to illness incidence, but also whether underlying factors continue to drive enduring impairment and poorer outcomes. We aim to extend the evidence on social inequalities and ethnic variation in outcomes after FEP, using large, national longitudinal dataset of patients receiving gold standard EIP care.

We wish to establish whether: (1) Black and Asian racial minority individuals with FEP differ in their long-term symptoms (psychosis symptoms and depression) and functional outcomes, compared to White individuals; (2) social deprivation contributes to later clinical and social outcomes across racial groups; (3) discharge services 2 years after EIS, differ by racial group.

## Method

### Study design

This was a secondary analysis of the National Evaluation of the Development and Impact of Early Intervention Services (NEDEN) study, a prospective longitudinal study of young people with a first episode of psychosis (FEP), across 14 early intervention services (EIS) in the UK [16]. The details of the original study methodology are reported elsewhere [16], but in brief, participants were initially recruited and assessed over the first 12 months of service as part of the NEDEN study. SUPEREDEN is the follow-on study, prospectively assessing the same cohort of individuals up until discharge from EIS (approximately at 3 years from baseline), and then up to 2 years post-discharge from EIS. Individuals with lived experience were involved in the study implementation and delivery and were regularly consulted throughout the SUPEREDEN project.

### Sample

The initial sample had a total of 978 participants. Participants were aggregated into 3 racial groups: a Black minority racial group (*n* = 71; 6.9%), Asian minority racial group (*n* = 157; 15%), and a White racial group (*n* = 750; 73%). The Black racial minority group included individuals who identified as Black Caribbean, Black African, and Black ‘other’. The White group included participants who identified as White British, White Irish or White ‘other’. The Asian group included participants who identified as Pakistani, Bangladeshi, Indian, or other Asian background. Participants met diagnostic criteria outlined in International Classification of Diseases under the following codes: F20, F25, F29, F31, F32–F32.1, and F32.3 [17]. Written and verbal consent was obtained for all participants. Ethical approval was given by Suffolk Local Research Ethics Committee, UK. REC reference number: 05/Q0102/44.

## Measures

### Outcome variables

Assessments were undertaken by research assistants who were trained and had no clinical involvement with the participants. A robust reliability protocol is detailed in the original research [16]. The following measures were used to assesses outcomes: Positive and Negative Syndrome Scale (PANSS) [18], Calgary Depression Scale for Schizophrenia (CDSS) [19], Global Assessment of Functioning Disability Scale (GAF Disability) [20], and Duration of Untreated Psychosis (DUP) [21].

### Covariate: social deprivation

A social deprivation proxy was derived at each time point by summing the presence of the following demographic factors: (1) unemployed, (2) single marital status, (3) living alone, and (4) living in temporary/supported accommodation or social housing, with each of these factors being assigned a score of 1 if present (maximum score = 4). A score of ‘1’ for living alone may also be indicative of financial stability or independence; however, a high score on our proxy measure (i.e., score of 4) is within the context of being unemployed, single and in supported or temporary accommodation, and hence more likely to signify social deprivation.

To validate the summation of these items, reliability statistics were inspected. Given that reliability coefficients such as Cronbach’s alpha are sensitive to the number of items in a scale and often lower with a smaller number of items, we interpreted this coefficient alongside the optimal mean inter-item correlations (*r* = 0.2–0.4), and explored the dimensionality of the data using a factor analysis [22, 23]. Correlations between items were significant (*p* < 0.01), and the mean inter-item correlations fell within the recommended range (*r* = 0.366), with a Cronbach’s alpha of 0.69 (Supplementary Material 1) [24]. The exploratory factor analysis confirmed the uni-dimensionality of the data, with all items loading strongly on a single component (Supplementary Material 1).

### Statistical analysis

#### Descriptive statistics

Chi-square tests for categorical, and between Analysis of Variance (ANOVA) tests for continuous variables were performed on the demographic, clinical, and social characteristics between racial groups at baseline and final follow-up (approximately 5 years from baseline).

#### Model building

To determine the longitudinal relationship between ethnic status and clinical and social outcomes, hierarchical linear mixed effect models were constructed within Statistical Package for the Social Sciences (SPSS v.25). Multi-level models were constructed in the following manner for PANSS Positive, PANSS Negative, PANSS General, GAF Disability, and Calgary Depression. At level 1, fixed and randomly varying time components were added to the model to examine the rate of change on the outcome for participants across the 5-year study period. Graphs were initially inspected to provide an indication of the shape of the growth trajectory alongside model fit indices to determine which rate of growth provided the best model fit. Lower scores on the Schwartz’s Bayesian Criterion indicated that a linear time component (coded as 0 for baseline and 1–4 for subsequent follow-ups) provided better model fit for each outcome and was therefore used to model the growth trajectories (Supplementary Material 2). At level 2, race was added to the covariance model to see if any variation in the (random) time slopes and intercepts for each of the outcomes were accounted for by racial group (Supplementary Material 2). At level 3, a social deprivation proxy was added as a covariate to determine its influence on the outcome when all variables were added (and controlled for) in the model. Models were estimated using a restricted maximum-likelihood (REML) method. REML was selected as it provides unbiased parameter estimates and is robust against large missing data and unbalanced designs [25–27]. Simulation studies have demonstrated that using REML to estimate the linear mixed models is preferable to multiple imputation for handling missing data when the mechanisms of missingness is assumed to be random; data imputation introduces greater noise into the models, rendering them more unstable [28, 29]. Results of the missing data analyses are reported on page 9. Finally, a diagonal covariance structure was used for the repeated and random effects which assumes heterogenous variances and no correlation between any of the elements [27].

#### Discharge services

A binary logistic regression was employed to explore the discharge destinations of the racial minority groups compared to the White racial group, 2 years following discharge from EIS. The binary outcome was coded as ‘1’ for secondary care (i.e., specialist mental health service support), or a ‘0’ for primary care (i.e., non-specialist community care from a general physician, on a needs basis). Electronic medical record data were accessed for this part of the analysis meaning that more complete (85.2%) data were obtained (*n* = 833; Black = 57; Asian = 147; White = 628).

## Results

### Sample description

#### Missing data

At baseline, outcome data were available for *n* = 912 participants (male = 632, 69.3%; mean age = 21.9 years), with an average retention rate of 33% (*n* = 296) by the final follow-up (5 years from baseline). This included data on 34% (*n* = 23) of the Black racial group, 37% (*n* = 52) of the Asian group, and 32% (*n* = 221) of the White group, by year 5. The greatest attrition was observed when participants reconsented into the SUPEREDEN study (Supplementary Material 3). To determine any bias in the patterns of missingness on the outcome variables, we conducted an exploratory analysis comparing individuals who remained in the study compared to those who did not. We did not find significant differences on any of the outcome measures at baseline (Supplementary Material 4), and there were no differences by racial group (*X*^2^ = 1.165, p = 0.559). We therefore assumed that missingness was not related to the outcomes of interest, and likely to be missing at random. A restricted maximum-likelihood method (REML) was considered appropriate to fit the linear mixed models [25, 28].

#### Demographic and clinical characteristics

At baseline and at 2 years post-discharge, there was a higher frequency of individuals within the Black racial group who were living alone, single, and living in temporary or supported accommodation (Table 1). They were also more likely to be unemployed at baseline, but there were no significant differences by follow-up. There were no significant differences in qualifications levels across racial groups.

**Table 1:** Demographic breakdown of racial groups at baseline and 2 years post-discharge from early intervention service

	Black *N* = 71	Asian *N* = 157	White *N* = 750	Statistical significance
Single marital status				
Baseline	66 (93%)	115 (73%)	660 (88%)	*p* < 0.001
2 years post-discharge	23 (79.3%)	25 (43.9%)	196 (71%)	*p* < 0.001
Living alone				
Baseline	15 (21%)	6 (3.8%)	106 (14.1%)	*p* < 0.001
2 years post-discharge	12 (42.9%)	5 (9.3%)	80 (31.9%)	*p* < 0.001
Unemployed				
Baseline	52 (73%)	102 (65%)	419 (56%)	*p* < 0.001
2 years post-discharge	21 (75%)	34 (63%)	149 (59.8%)	NS
Qualifications				
None	18 (27%)	42 (28%)	168 (23%)	NS
GSCE/NVQ	32 (48%)	59 (39%)	293 (40%)	
A-level/BTEC	17 (25%)	39 (26%)	192 (26%)	
Degree	4 (6%)	12 (8%)	79 (11%)	
Place of birth: UK	40 (62%)	121 (79%)	726 (97%)	*p* < 0.001
Housing type				
Owned/parents own	25 (36%)	117 (76%)	396 (56%)	*p* < 0.001
Rented	34 (49%)	24 (16%)	250 (35%)	
Temporary or supported	11 (15%)	13 (8%)	66 (9%)	

*NS* non-significant

The clinical characteristics of the sample are provided in Table 2. There were no significant differences across the groups with age of onset; however, the White group had a significantly longer median DUP, and a significantly higher percentage of the White racial group had reported self-harm and used cannabis persistently. There were no significant differences between the racial groups on medication adherence and prescriptions of clozapine or psychological therapies (Table 2). Over the follow-up period, the Black racial group had a higher average score on our proxy measure of deprivation (*b* = 0.406, *p* < 0.001, 95% CI [0.187, 0.624]), whilst the Asian group had a lower score (*b* = − 0.322, *p* < 0.001, 95% CI [− 0.477, − 0.168]) compared to the White group.

**Table 2:** Clinical characteristics across racial groups

	Black *N* = 71	Asian *N* = 157	White *N* = 750	Statistical significance
Presentation factors				
Delay of untreated psychosis (weeks; median)^a^	6.43	8.64	12.71	*p* < 0.05
Age of onset (years; mean/SD)	21.72 (4.7)	21.05 (4.17)	21.4 (5.17)	NS
Ongoing factors				
Cannabis use^b^ (persistent)	3 (4.2%)	8 (5.5%)	107 (14.7%)	*p* < 0.001
Self-harm (*n*; %)^c^ Yes/no	1; 53 (1.9%)	5; 117 (4.1%)	85; 510 (14.3%)	*p* < 0.002
Treatment factors				
Medication Non-adherence^d^	10 (14.1%)	22 (14%)	89 (11.9%)	NS
Clozapine^e^	2 (2.8%)	8 (5.1%)	14 (1.86%)	NS
Psychological therapy^f^	13 (18.3%)	28 (17.8%)	125 (16.7%)	NS

*NS* non-significant

^a^Independent median test

^b^Persistent cannabis use = continued cannabis use over 12 months derived from the Drug Check [30]

^c^Client reported self-harm; any incidence of self-harm over the initial 12 months of treatment

^d^Medication adherence derived as an average score from the clinician-rated ‘Service Engagement Scale’ [31]

^e^Prescribed clozapine within the first year of EIS treatment

^f^Received an individualised form of therapy, e.g., cognitive behavioural therapy across the full study period

### Racial group differences on recovery outcomes

#### Linear time effect (level 1)

Over the follow-up period, there were significant main effects of time for PANSS positive, PANSS negative, PANSS general, and Calgary Depression, with symptoms decreasing on average over the follow-up period. GAF disability scores on average increased over the study period, with higher scores indicating improved functioning (Table 3).

**Table 3:** Linear mixed model fixed effects analysis of recovery outcomes over the 5-year study period

	Beta	SE	*p* value	Lower-95	Upper-95
PANSS positive	− 0.502	0.077	< 0.001	− 0.652	− 0.352
PANSS negative	− 0.335	0.071	< 0.001	− 0.474	− 0.197
PANSS general	− 1.037	0.126	< 0.001	− 1.284	− 0.789
CDSS	− 0.473	− 0.065	< 0.001	− 0.600	− 0.345
GAF disability	1.582	0.243	< 0.001	1.104	2.060

*PANSS* Positive and Negative Syndrome Scale, *CDSS* Calgary Depression Syndrome for Schizophrenia

#### Illness trajectories and race (level 2)

**Fig. 1:**
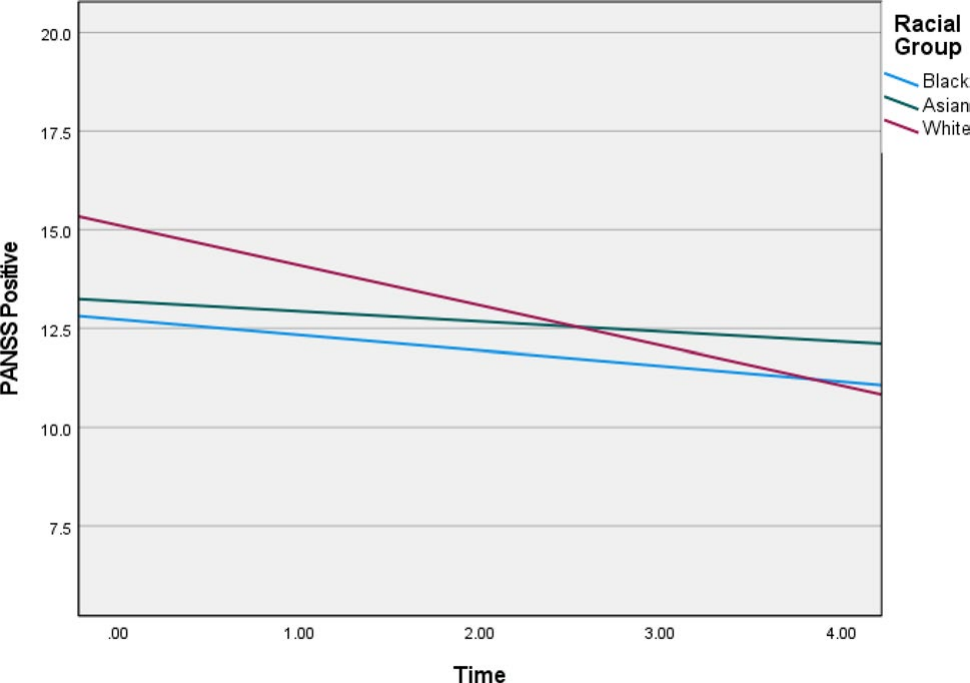
Graphs depicting illness trajectories across the racial groups on PANSS positive over the 5-year follow-up

**Fig. 2:**
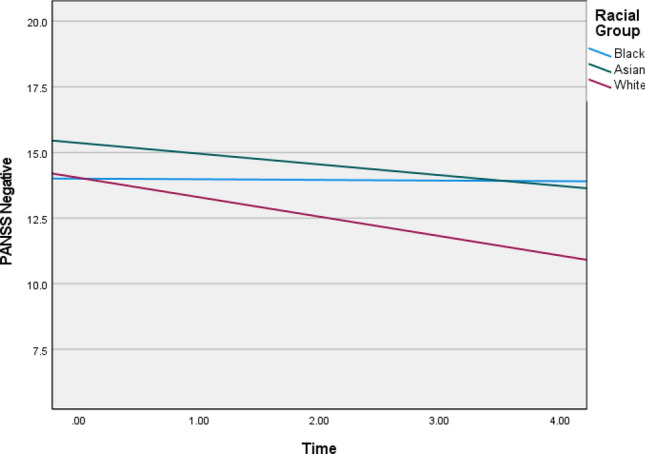
Graphs depicting illness trajectories across the racial groups on PANSS negative over the 5-year follow-up

**Fig. 3:**
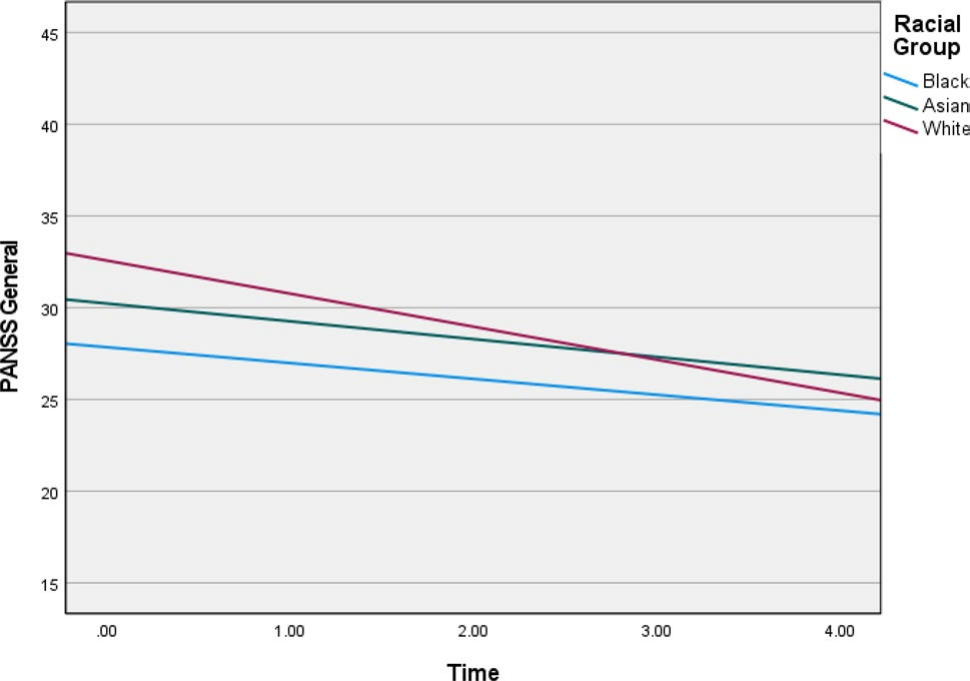
Graphs depicting illness trajectories across the racial groups on PANSS general symptoms over the 5-year follow-up

**Fig. 4:**
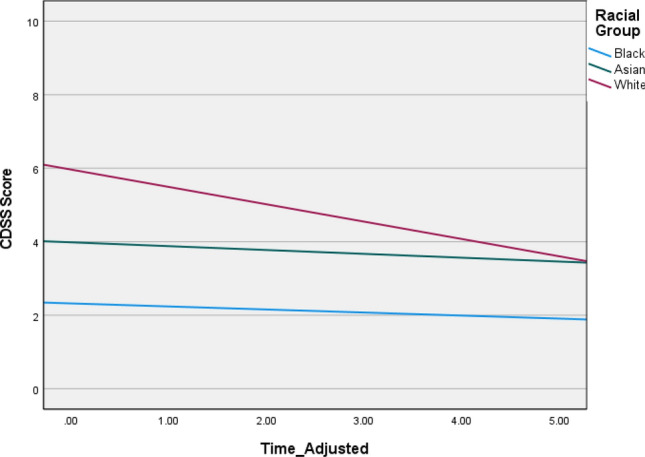
Graphs depicting illness trajectories across the racial groups on CDSS depression symptoms over the 5-year follow-up

**Fig. 5:**
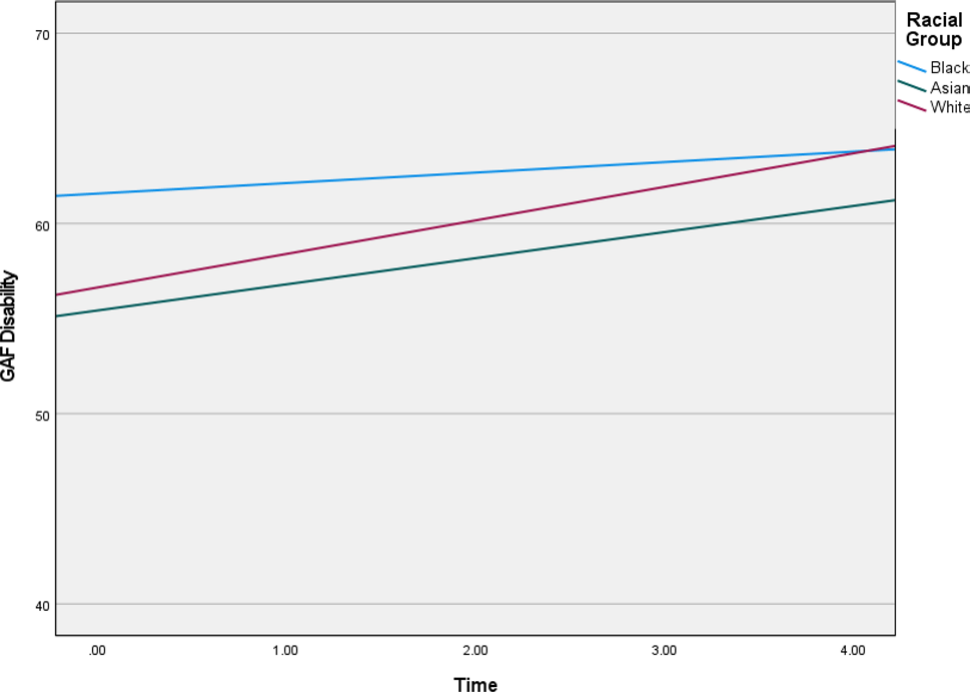
Graphs depicting illness trajectories across the racial groups on GAF disability over the 5-year follow-up

The random covariance analysis indicated significant variation in the intercepts and linear slopes across the racial groups for PANSS positive (*b* = 0.140; 95% CI [0.679, 1.235]), negative (*b* = 0.497; 95% CI [0.315, 0.783]), and general symptoms (*b* = 2.593; 95% CI [1.961, 3.428]), as well as GAF disability (*b* = 14.078, 95% CI [11.212, 17.677]) and depression (*b* = 0.684; 95% CI [0.261, 0.648]). The growth trajectories are summarised in Table 4, and visualisation of the trajectories are provided in Figs. 1, 2, 3, 4 and 5 (see also Supplementary Material 5 for means and standard deviations). Steeper slopes were observed for the White racial group. The Black group showed no significant variation in growth for PANSS positive and Calgary Depression. Lower symptom scores were observed for the Black group at baseline, whilst the White group had higher scores, except for negative symptoms, where the Asian group were observed to have higher scores at baseline.

**Table 4:** Linear growth parameters across racial groups over time for each of the outcome variable

	Beta	Wald *Z*	*p *value	Lower-95	Upper-95
PANSS positive					
Intercept^a^	4.824	4.894	< 0.001	3.232	7.200
Group × time^b^					
Black	0.706	1.935	*NS*	0.257	1.944
Asian	1.372	3.4943	< 0.001	0.783	2.406
White	1.121	6.656	< 0.001	0.835	1.505
PANSS negative					
Intercept^a^	7.275	6.568	< 0.001	5.398	9.805
Group × time^b^					
Black	1.541	2.500	0.0124	0.704	3.374
Asian	0.929	2.838	0.0045	0.466	1.85
White	0.609	4.487	< 0.001	0.394	0.943
PANSS general	11.554^a^	4.381	< 0.001	7.387	18.073
Group × time^b^					
Black	2.988	2.418	0.016	1.329	6.721
Asian	2.695	3.175	0.002	1.454	4.997
White	2.944	6.784	< 0.001	2.205	3.930
CDSS					
Intercept^a^	6.1502^a^	8.770	< 0.001	4.9185	7.690
Group × time^b^					
Black	0.074	0.424	*NS*	0.001	7.561
Asian	0.434	2.214	0.033	0.1791	1.053
White	0.812	6.406	< 0.001	0.598	1.103
GAF disability					
Intercept^a^	81.516	9.351	< 0.001	66.103	100.524
Group × time^b^					
Black	8.537	1.986	0.0471	3.182	22.909
Asian	12.125	3.530	< 0.001	6.959	21.127
White	14.933	7.8943	< 0.001	11.650	19.142

^a^Random covariance parameter for the intercepts across racial groups

^b^Random covariance slope parameter for time × racial group

*NS* non-significant at 0.05 alpha level

#### Social deprivation, race, and outcome (level 3)

A ‘social deprivation proxy’ was added as a covariate in the linear mixed models for each of the outcome variables described above. Social deprivation proxy score significantly contributed to variance in outcomes across the racial groups. Higher scores on the social deprivation proxy was associated with higher PANNS positive scores (*b* = 0.710, SE = 0.103, *p* < 0.001, 95% CI [0.509, 0.912]), PANSS negative scores, *b* = 0.875, SE = 0.106, *p* < 0.001, 95% CI [0.667, 1.083]), PANNS general score (*b* = 1.390; SE = 0.174, *p* < 0.001, 95% CI [1.050, 1.731]), and Calgary depression scores (*b* = 0.455, SE = 0.099, *p* < 0.001, 95% CI [0.261, 0.648]). Finally, a higher social deprivation score on our proxy measure was associated with lower GAF scores (*b* = − 5.116, SE = 0.328, *p* < 0.001, 95% CI [− 5.758, − 4.473]).

### Discharge trajectories

A binary logistic regression comparing discharge services across racial groups 2 years following EIS, showed that, compared to their White counterparts, there was a greater likelihood for the Asian (OR = 3.04; 95% CI [2.050, 4.498]; *p* =  < 0.001) and Black racial group (OR = 2.47; 95% CI [1.354, 4.520]; *p* =  < 0.001) to remain in secondary care (i.e., treatment within mental health services) by follow-up.

## Discussion

In this large, prospective FEP cohort, recovery outcomes significantly improved across the follow-up period, which included the duration of EIS care and up to 2 years post-discharge.

The rate of improvement varied by racial group, with the White group showing more growth in their recovery trajectories. Social deprivation further contributed to this variance in growth across racial groups. Two years following EIS care, the Asian and Black individuals were less likely to be discharged from mental health services.

To our knowledge, this is the first study to report long-term outcomes across different racial minority groups following EIS care [6, 11–15]. Our findings hint at the potential compounded impact of the intersectional challenges of racial minority status and deprivation [6, 12, 32–34]. However, our findings are nuanced; deprivation was not uniform across minority racial groups. The Black group had significantly greater levels of deprivation, whilst the Asian group experienced less social deprivation over the study period.

Despite improving more, the White group typically had similar levels of symptoms to the minority racial groups by follow-up, possibly suggesting a ceiling effect in recovery trajectories for the minority racial groups. This was likely the case for the Black group who showed no change in growth over time on the Calgary Depression Scale, but this was in the context of low, stable symptoms across the time frame. Similarly, self-harm was less frequent in the Black group; a finding supported by previous research [6]^6^.

Nevertheless, we showed that minority individuals were more likely to be receiving mental health treatment following discharge from EIS, suggesting that they may not have achieved the same level of recovery as their White counterparts. This possible enduring nature of psychosis for minority groups would support the work of Morgan et al., where Black individuals were more likely to have a continuous, non-remitting illness course, as opposed to an episodic trajectory [6].

### Confounding treatment factors

It is well documented that a prolonged delay of untreated psychosis (DUP) is associated with poorer recovery outcomes [35, 36]. We did not find a longer DUP for racial minority groups. Instead, the White group had a significantly longer DUP; a finding supported by other studies [37–40]. This may account for the higher symptom scores for the White group at baseline, yet the White group typically showed more growth in their trajectories over time. This raises the question as to why the trajectories of the racial minority groups may appear less responsive to the support offered within current service models.

Linked to this notion, our initial inspection showed no differences in treatment factors that are likely to influence recovery outcomes, such as medication adherence, treatment with clozapine, and receiving a psychological therapy [41–43]. Further, we found no differences in age of onset of illness, but there were significant differences in persistent cannabis use, which was more frequent in the White group. However, as previously reported by the EDEN consortium, the influence of cannabis on poor outcomes was shown to be independent of ethnic status [44].

### Proposed mechanisms driving inequalities in outcomes

Socio-economic status, experiences of racism, linguistic distance, and social exclusion and discrimination may lead to a psychological ‘disempowerment’ [45] or ‘social defeat’ [46]. Such processes are likely to play an important role in the aetiology and pathogenesis of psychosis [34, 46, 47]. Indeed, in our study, for the Black racial group, deprivation was already apparent at baseline, likely reflecting a longstanding trajectory of deprivation. This not only exposes these individuals to psychotic illness, but is likely to be mutually reinforcing, where psychosis symptoms drive further deprivation and exclusion, and vice versa, resulting in enduring impairment, marginalisation, and further feelings of disempowerment [6, 33, 48].

On the other hand, the Asian minority group experienced less deprivation compared to the other groups, which suggests that other factors are also likely to play a part. Indeed, previous studies have reported racial-ethnic differences in receiving evidence-based interventions and family psychoeducation once in treatment following a first episode of schizophrenia [49]. Compulsory treatment is also frequently reported [9]. Themes of mistrust in services, stigma, and coerciveness have also featured in the narratives of Black and minority individuals receiving mental health treatment [50]. Thus, treatment trajectories, including pathways out of EIS, warrant in-depth exploration, particularly as our racial minority groups were less likely to be discharged out of mental health service 2 years following EIS discharge. The lived experiences of these individuals will be essential to fully understand the processes behind these disparities.

### Strengths and limitations

There are several strengths to this study. The EDEN studies comprised a large, perspective cohort of participants who had experienced FEP across distinct and varied geographical areas in England, making it representative of the UK’s diverse population, but also representing socioeconomic variability. We add to past literature by further including a comparison with individuals of Asian heritage, which has not been robustly reported within the literature. Finally, we explore a range of outcome variables and model the heterogeneity in illness trajectories across the duration of EIS care and the subsequent 2 years following discharge. However, there are important study limitations to consider.

First, whilst over a thousand participants originally consented to the study, our target minority racial groups were substantially smaller, reducing our statistical power. Given the high prevalence of psychosis within ethnic minority groups in high-income countries, our small group size in this study may reflect lack of engagement of minority individuals in research, thus placing limit on the representativeness of our findings and potentially biasing the sample. Second, there were high levels of attrition across each time point, potentially introducing bias in our findings. We were, however, able to demonstrate that missingness did not differ by racial group, and there were no differences by racial group on the main outcomes at baseline for those who continued in the study compared to those who dropped out. In such situations where mechanisms of missingness are assumed to be random, the REML algorithm (used within the analysis) is shown to be robust to large missing data and unbalanced designs [25, 28, 29]. Third, for reasons of statistical power, we were not able to explore intergroup differences. For example, there is evidence pointing to differential outcomes in Black Caribbean, as opposed to Black African individuals [6, 51]. We also did not include a mixed racial group in our analysis because of the limited sample size; this should be investigated further. Finally, as this was a secondary analysis of existing data, this restricted our examinations into other potential factors influencing the observed differences. This also meant that a proxy estimate was used to quantify social deprivation. Future research may wish to build on these findings using a more robust measure of social deprivation, which also considers the premorbid levels of deprivation, compared with the deprivation synergistically linked to psychosis.

## Implications and future directions

Methodological issues place limit on how much we can extrapolate our findings, but they nevertheless add to a growing body of research indicating differential outcomes for racial minorities recovering from a first episode psychosis. In addition to replication, further research is also needed to understand the key drivers of these disparities that may serve as pivotal points for intervention. Our findings may suggest wider contextual and societal factors feeding into illness trajectories. Systemic barriers and social structures inherent within our society are likely to permeate into health care and place limit on one’s outcome. Breaking this cycle should not only be a priority for EIS, but a shared priority for public health and social policy [6].

There is growing interest looking into area-level interventions to mitigate the psychological consequences of belonging to a disempowered minority group. For example, increasing access to social capital is proposed to dampen the social stress associated with deprivation and discrimination, and thus foster an environment that is more conducive to recovery [11, 52, 53]. Though promising, implementing such interventions is complex given their nuanced and context-dependent nature [54]. At a service level, there may be a need to develop clinicians’ cultural competencies, in addition to offering culturally sensitive interventions to improve service provision for underserved groups. Co-produced work will be an important step towards achieving this goal [55]. Finally, exploring the disempowerment experienced by such individuals may also be an important target for clinical intervention [47].

## Conclusion

In a large FEP cohort, our findings suggest variations in long-term clinical and social outcomes following EIS for racial minority groups. Social deprivation contributed to this variance, with Black individuals experiencing the most deprivation. Black and Asian individuals were also less likely to be discharged from mental health service by follow-up. Though replication is needed, our findings hint at the need for targeted, and culturally sensitive service provision, that mitigates the impact of discrimination and deprivation and promotes long-term recovery following FEP.

## Supplementary Information

Below is the link to the electronic supplementary material.

Supplementary file1 (DOCX 25 KB)

Supplementary file2 (DOCX 25 KB)

Supplementary file3 (DOCX 62 KB)

Supplementary file4 (DOCX 23 KB)

Supplementary file5 (DOCX 36 KB)

## Data Availability

The datasets generated during and/or analysed during the current study are not publicly available under current ethical approvals but are available from the corresponding author on reasonable request.
