# Supplementary material for: Five-year illness trajectories across racial groups in the UK following a first episode psychosis
Source: Soc Psychiatry Psychiatr Epidemiol. 2023 Jan 30;58(4):569–79. doi: 10.1007/s00127-023-02428-w (PMC10066114; doi:10.1007/s00127-023-02428-w)
Supplement: Supplementary file 1 — Supplementary file1 (DOCX 25 KB) [file 127_2023_2428_MOESM1_ESM.docx]

Supplementary Material – 1

*Statistical Validation of the Social Deprivation Proxy Measure*

1. Inter-item Correlations

|  | Single | Unemployed | Living Alone | Temporary, Social or Supported Hosing |
| --- | --- | --- | --- | --- |
| Single | ------- | 0.405^**^ | 0.306^**^ | 0.258^**^ |
| Unemployed |  | ------- | 0.347^**^ | 0.457^**^ |
| Living Alone | 0.306^**^ | 0.347^**^ | ------- | 0.418^**^ |
| Temporary, Social or Supported Hosing | 0.258^**^ | 0.457^**^ | 0.418^**^ | ------- |

^**^Correlation is significant at the 0.01 level (2-tailed).

1. Exploratory Factor Analysis

| Item | Factor Loadings |
| --- | --- |
| Unemployed | 0.777 |
| Housing | 0.747 |
| Living Alone | 0.712 |
| Single | 0.659 |
|  |  |
| Eigenvalue | 2.101 |
| % of Variance | 52.53% |

*Extraction method: Principal Component Analysis*
