# Supplementary material for: Five-year illness trajectories across racial groups in the UK following a first episode psychosis
Source: Soc Psychiatry Psychiatr Epidemiol. 2023 Jan 30;58(4):569–79. doi: 10.1007/s00127-023-02428-w (PMC10066114; doi:10.1007/s00127-023-02428-w)
Supplement: Supplementary file 2 — Supplementary file2 (DOCX 25 KB) [file 127_2023_2428_MOESM2_ESM.docx]

Supplementary Material 2

**Model fit indices for growth trajectories across each recovery outcomes**

| **Model Type** | **Schwarz's Bayesian Criterion (BIC)^*^** |
| --- | --- |
| PANSS Positive |  |
| Quadratic Growth | 15160.169747 |
| Linear Growth | 15048.998582 |
| Covariate: Racial Group^a^ | **15026.659763** |
| PANSS Negative |  |
| Quadratic Growth | 15042.491334 |
| Linear Growth | 14998.514104 |
| Covariate: Racial Group^a^ | **14983.542048** |
| PANSS General |  |
| Quadratic Growth | 17294.069405 |
| Linear Growth | 17217.035529 |
| Covariate: Racial Group^a^ | **17209.652255** |
| Calgary Depression Scale |  |
| Quadratic Growth | 16055.827190 |
| Linear Growth | 16010.661371 |
| Covariate: Racial Group^a^ | **15966.633773** |
| GAF Disability Scale |  |
| Quadratic Growth | 23095.359964 |
| Linear Growth | 23018.296496 |
| Covariate: Racial Group^a^ | **22787.424469** |

^*^Schwarz's Bayesian Criterion (BIC) – Lower scores indicate better model fit. ^a^ Covariate added to the Linear growth model.
