# Supplementary material for: Five-year illness trajectories across racial groups in the UK following a first episode psychosis
Source: Soc Psychiatry Psychiatr Epidemiol. 2023 Jan 30;58(4):569–79. doi: 10.1007/s00127-023-02428-w (PMC10066114; doi:10.1007/s00127-023-02428-w)
Supplement: Supplementary file 3 — Supplementary file3 (DOCX 62 KB) [file 127_2023_2428_MOESM3_ESM.docx]

Follow-up 1: Year 1 EIS

White = 579

Follow-up 1: Year 1 EIS

Black = 50

Follow-up 1: Year 1 EIS

Asian = 114

Follow-up 2: Year 3 EIS

White = 357

Follow-up 2: Year 3 EIS

Asian = 67

Follow-up 2: Year 3 EIS

Black = 33

Follow-up 4: Year 2 post EIS discharge

Black = 23

Follow-up 4: Year 2 post EIS discharge

Asian = 52

Follow-up 4: Year 2 post EIS discharge

White = 221

Follow-up 3: Year 1 post EIS discharge

White = 267

Follow-up 3: Year 1 post EIS discharge

Asian = 47

Follow-up 3: Year 1 post EIS discharge

Black = 21

*Supplementary Material 3. Participant flow through the study on available outcome data by racial group.*
