# Supplementary material for: Five-year illness trajectories across racial groups in the UK following a first episode psychosis
Source: Soc Psychiatry Psychiatr Epidemiol. 2023 Jan 30;58(4):569–79. doi: 10.1007/s00127-023-02428-w (PMC10066114; doi:10.1007/s00127-023-02428-w)
Supplement: Supplementary file 4 — Supplementary file4 (DOCX 23 KB) [file 127_2023_2428_MOESM4_ESM.docx]

Supplementary Material 4.

**Drop-outs analysis comparing the main outcomes at baseline for participants who re-consented into the SUPEREDEN study versus those who did not.**

|  | *F statistic* | *P* Value | Effect Size  Lower-95 | Effect Size  Upper-95 |
| --- | --- | --- | --- | --- |
| PANSS Positive | 0.478 | 0.490 | 0.000 | 0.008 |
| PANSS Negative | 2.007 | 0.157 | 0.000 | 0.013 |
| PANSS General | 0.024 | 0.877 | 0.000 | 0.004 |
| CDSS | 1.118 | 0.291 | 0.000 | 0.001 |
| GAF Disability | 0.000 | 0.998 | 0.000 | 0.000 |

*Note: df = degrees of freedom; F-statistic derived from a One-way Analysis of Variance (ANOVA); Effect size = Eta Squared.*
