# Supplementary material for: Five-year illness trajectories across racial groups in the UK following a first episode psychosis
Source: Soc Psychiatry Psychiatr Epidemiol. 2023 Jan 30;58(4):569–79. doi: 10.1007/s00127-023-02428-w (PMC10066114; doi:10.1007/s00127-023-02428-w)
Supplement: Supplementary file 5 — Supplementary file5 (DOCX 36 KB) [file 127_2023_2428_MOESM5_ESM.docx]

|  | *Ethnicity* | *1* |  | *2* |  | *Time*  *3* |  | *4* |  | *5* |  |
| --- | --- | --- | --- | --- | --- | --- | --- | --- | --- | --- | --- |
|  |  | *M* | SD | M | SD | M | SD | M | SD | M | SD |
| PANSS Positive | Black  Asian  White | 12.41  13.15  15.89 | 5.87  6.22  5.83 | 10.58  11.26  11.34 | 5.74  4.78  4.45 | 11.61  13.13  12.33 | 4.94  6.27  4.87 | 11.00  11.98  12.24 | 4.37  4.55  5.12 | 11.35  12.21  12.16 | 4.64  5.27  5.19 |
| PANSS Negative | Black  Asian  White | 14.08  16.24  14.49 | 5.75  7.52  6.29 | 13.50  13.81  11.51 | 6.48  5.83  5.25 | 14.53  15.77  12.04 | 5.18  6.27  5.07 | 11.60  13.74  11.60 | 3.87  5.23  4.52 | 15.04  14.25  11.98 | 6.18  4.78  4.76 |
| PANSS General | Black  Asian  White | 27.73  30.66  33.63 | 8.82  10.53  9.67 | 23.22  36.23  26.11 | 7.67  8.76  8.14 | 24.32  29.15  28.32 | 6.96  9.26  8.28 | 23.67  26.38  27.06 | 7.34  7.74  8.41 | 26.13  26.98  26.88 | 8.63  8.02  8.30 |
| CDSS | Black  Asian  White | 2.67  4.30  7.08 | 3.97  4.56  5.42 | 1.45  3.01  3.86 | 2.64  4.27  4.50 | 0.81  4.08  3.65 | 4.01  4.78  4.97 | 2.35  3.70  4.25 | 4.13  4.38  4.76 | 1.86  3.48  4.25 | 3.30  4.05  4.62 |
| GAF Disability | Black  Asian  White | 58.61  53.73  52.98 | 15.36  15.70  15.34 | 66.16  60.84  63.89 | 18.95  17.28  16.89 | 65.79  57.02  63.14 | 13.70  16.78  16.95 | 62.35  60.00  60.52 | 14.64  17.52  17.53 | 59.50  59.95  60.16 | 14.21  17.13  18.43 |

Supplementary Material 5

**Means and Standard Deviations across racial groups for each of the outcome variables over the follow-up period.**

M = Mean; SD = Standard Deviation.
